# Horseradish Peroxidase-Catalyzed Degradation of Reactive Blue 171 Dye

**DOI:** 10.1021/acsomega.5c01374

**Published:** 2025-05-26

**Authors:** Rafael Faria Giovanella, Winnie Brandão Queiroz, Jürgen Andreaus, Endler Marcel Borges, Paulo Cesar de Jesus

**Affiliations:** Departamento de Química, 37874 Universidade Regional de Blumenau, Antonio da Veiga, 140, Blumenau, SC 89030-903, Brazil

## Abstract

Despite establishing covalent bonds with cellulose, significant amounts of reactive dyes remain unfixed and are lost through secondary reactions during cellulosic fiber dyeing, ultimately entering textile wastewater and making additional treatment necessary. In this work, the kinetics of the biocatalytic degradation of the textile dye Reactive Blue 171 with horseradish peroxidase (HRP PeO 906) with an activity of 2009.2 kU/g was studied. The influence of temperature (in the range of 30–70 °C), pH (in the range of 4–8), the amount of biocatalyst (1, 2, and 3 mg), and hydrogen peroxide concentration (up to 0.3%) used in the process was evaluated. Biodegradation was monitored by the disappearance of the maximum absorption band of the dye at 625 nm. Biodegradation of Reactive Blue 171 followed first-order kinetics with rate constants ranging from 1.538 × 10^–2^ min^–1^ (30 °C) to 7.514 × 10^–2^ min^–1^ (70 °C) and *R* ^2^ ≥ 0.998. The best result observed for the biodegradation of Reactive Blue 171 was obtained at 40 °C when 0.1 g L^–1^ of dye, 3 mg of enzyme, and 0.3% H_2_O_2_ were used. Under these conditions activation parameters were determined as *E*_a_ = 13.24 kJ mol^–1^(*r* ^2^ = 0.9919), Δ*H* ^#^ = 10.55 kJ mol^–1^ (*r* ^2^ = 0.9717), Δ*G* ^#^_medium_ = 14.09 kJ mol^–1^, and Δ*S* ^#^_medium_ = 0.109 kJ K^–1^ mol^–1^, achieving a decolorization of 81.40% after 120 min. Biodegradation involved low energy variation and was favored by increases in temperature and biocatalyst concentration. In the investigated pH range, the highest dye degradation was observed at pH 4 and 5. The obtained decolorization results of Reactive Blue 171 with HRP and H_2_O_2_ indicate that the biodegradation of textile dyes is a viable and sustainable method.

## Graphical abstract

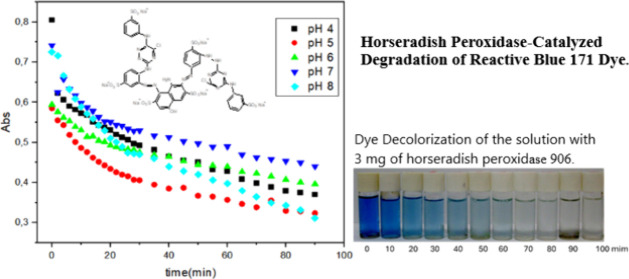

## 1 Introduction

As the global population continues to grow, wastewater volumes are rising, leading to higher pollution levels. Although treatment plants manage industrial discharges, they often fail to effectively eliminate many organic contaminants from sources such as industries, hospitals, and agriculture. Consequently, researchers are increasingly exploring alternative methods for pollutant removal.^1–3^

The textile industry is one of the largest consumers of water and synthetic dyes, discharging substantial volumes of complex effluents enriched with high organic loads and elevated levels of inorganic salts. Given that most dyes in commercial use are synthetic, their widespread and growing application across various processes has become a source of increasing environmental concern. A growing body of research indicates that many of these compounds exhibit toxic and potentially carcinogenic properties, reinforcing the critical need for advanced and sustainable wastewater treatment technologies.^4^

The complexity of textile effluents coupled with increasingly stringent environmental regulations has driven the pursuit of advanced treatment technologies capable of effectively removing toxic organic pollutants through degradation or adsorption. Optimal solutions must balance cost-effectiveness, operational efficiency, and treatment time, while ensuring thorough detoxification and facilitating the potential reuse of treated wastewater in industrial processes.^5^

The degradation of pharmaceuticals and dyes is often catalyzed by nanocomposites and photocatalysis. For example, captopril was degraded using ozonation catalyzed by a ZnO/Fe_2_O_3_ nanocomposite.^6^ Methylene blue, methyl orange, methyl violet, rhodamine B, basic fuchsin, and thymolphthalein were degraded using a ZnO-coated graphene oxide (ZnO/GO) nanocomposite under ambient light.^7^ The photo-Fenton oxidation of methylene blue was catalyzed by iron nanocomposites.^8^

Degradation of azo dyes by graphene-loaded TiO_2_ nanocomposites was described.^9^ Methyl orange was degraded by photocatalytic reaction under UV and visible light using a Pt-modified TiO_2_ nanocomposite.^10^ The degradation of rhodamine B dye was catalyzed by an iron nanocomposite under ultrasonic radiation, using H_2_O_2_ as the oxidant.^11^

Sarafloxacin was degraded through ozonation catalyzed by modified activated carbon.^12^ A graphene oxide-based membrane modified with iron nanocomposites was found to enhance photo-Fenton catalytic degradation of dyes.^13^ Methylene blue was degraded under sunlight irradiation, catalyzed by graphene/TiO_2_ nanocomposites.^14^

Anodic oxidation has also been explored for the degradation of dyes, pharmaceuticals, and pesticides, with electrode doping using PbO_2_ as an interesting alternative.^15,16^

Advanced oxidation processes have been presented as efficient alternatives for the treatment of effluents using oxidation reactions involving hydroxyl (OH^·^) or sulfate (SO_4_^2·–^) radicals among other species. The main advantage of these processes, which have been extensively studied and used in the removal of dyes and the treatment of effluents from textile industries, is the complete destruction of organic contaminants, converting them into carbon dioxide, water, and inorganic salts.^17^

Among the various technologies investigated for textile dye removal, biocatalytic degradation has emerged as a particularly promising and green approach.^18^ A diverse range of microorganisms, including fungi, yeasts, enzymes, and algae, have been employed for the biological degradation of dyes.^19^ The degradation of azo dyes—through chemical, enzymatic, or combined methods—has been extensively studied in recent years.^20^

The biodegradation of 8-anilino-1-naphthalenesulfonic acid—a precursor commonly used in the synthesis of azo dyes—was investigated using *Pseudomonas aeruginosa*. Degradation initiated during the exponential growth phase of the microorganism. High-performance liquid chromatography (HPLC) and gas chromatography–mass spectrometry analyses revealed salicylic acid and keto-α-adipic acid as major metabolic intermediates.^21^

Spectrophotometry and HPLC techniques were employed to identify potential intermediates in the degradation of Disperse Yellow 3 and Disperse Orange 3 by the fungus Pleurotus ostreatus. The results highlight the potential of the fungus for the effective removal of disperse dyes from effluents.^22^

Fungi have been the subject of various studies on the biodegradation of textile dyes,^23^such as white rot fungi^24^ for reactive dyes or Trichophyton rubrum and other fungi for the degradation of azo dyes.^25^

In the presence of hydrogen peroxide, studies on the biodegradation of Remazol Brilliant Blue R using ligninolytic enzymes have demonstrated optimal degradation performance, even under varying conditions.^26^

At pH 7, *C.I. Reactive Blue 13* was degraded using an isolated Pseudomonas sp. strain in an anaerobic/aerobic system, achieving 83.2% decolorization.^27^ With the fungus Lentinus crinitinus, 95% degradation of *C.I. Reactive Blue 220* was obtained.^27^ Additionally, *C.I. Reactive Red 195* was degraded in an alternating anaerobic/aerobic system by a bacterial consortium, reaching 97% efficiency, as confirmed by the complete disappearance of color in the solution.^28^

The white rot fungi Schizophyllum sp. F17 were tested for the degradation of Congo red, Alizarin red, Neutral red, and Crystal violet, and they exhibited decolorization efficiencies of 92.4, 93.4, 83.6, and 70.5%, respectively, within 12 h.^29^ The degradation of the dye Remazole Blue Turquoise G133 with soy peroxidase/H_2_O_2_ achieved an efficiency of 96%, highlighting biocatalysis as a promising method for dye degradation.^30^

Horseradish peroxidase (HRP) catalyzed the decolorization of aqueous Indigo Carmine solutions within a temperature range of 25–60 °C, following first-order kinetics. Under acidic to neutral conditions (pH 4, 5, and 6), temperatures between 40 and 60 °C, and hydrogen peroxide concentrations between 0.3 and 0.003%, the use of 2 mg of HRP resulted in up to 99% decolorization. These findings demonstrated that HRP combined with H_2_O_2_ is an effective catalytic system for the decolorization and degradation of Indigo Carmine dye.^31^ Based on these results, the same biocatalyst was applied in this study to Reactive Blue 171.

## 2 Experimental Section

### 2.1 Materials

HRP PeO 906, kindly provided by Toyobo do Brasil Ltd. (São Paulo, Brazil), had an enzymatic activity of 2009.2 kU/g and was used without further modification. Hydrogen peroxide (H_2_O_2_) 30% was purchased from VETEC (Brazil) and diluted to final concentrations of 0.03, 0.3, and 3%.

Reactive Blue 171, with a molecular weight of 1433.95 g/mol and a maximum absorption at λ_max_ = 625 nm, was kindly supplied by Siderquímica (São José dos Pinhais, Paraná, Brazil). Its chemical structure is shown in Figure 1.

**Figure 1:**
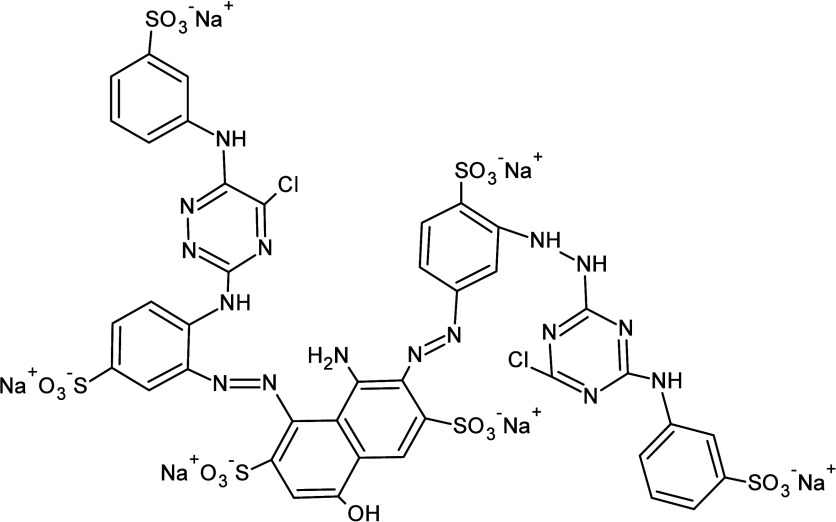
Structure of the dye C.I. Reactive Blue 171.

Solutions with varying pH values were prepared using McIlvaine buffer, composed of sodium dihydrogen phosphate (Na_2_HPO_4_) and citric acid (C_6_H_8_O_7_), both obtained from VETEC.

The experiments were monitored by measuring the absorbance of the dye at its maximum absorption wavelength using a CARY 50 BIO UV/vis spectrophotometer (Varian) equipped with a thermostat for temperature control.

### 2.2 Solutions

A dye solution with a known concentration of 0.1 g L^–1^ Reactive Blue 171 was prepared with distilled water. For the biocatalytic degradation studies, 1 mL of hydrogen peroxide at concentrations of 0.3 and 0.03% was used, along with varying amounts of the enzyme HRP PeO 906:1.0, 2.0, and 3.0 mg (±0.0001 g).

The initial unbuffered Reactive Blue 171 solution had a pH of 5. To assess the influence of pH on the oxidative degradation process, buffer solutions with pH values of 4.0, 5.0, 6.0, 7.0, and 8.0 were prepared using 0.1 mol L^–1^ citric acid and 0.1 mol L^–1^ disodium phosphate (McIlvaine buffer).

### 2.3 Procedures

Dye degradation experiments were performed in a 125 mL Erlenmeyer flask, where 1 mL of H_2_O_2_ and 25 mL of dye solution were added, along with varying amounts of solid HRP (1, 2, or 3 mg). The reaction system was agitated continuously in a Dubnoff-type water bath equipped with a thermostated pendulum stirrer. At regular intervals, 3 mL aliquots were taken with a pipet and transferred directly into a quartz cuvette for absorbance measurement at the maximum wavelength of Reactive Blue 171 dye (λ_max_ = 625 nm).

The temperature was varied in 10 °C increments from 30 to 70 °C. Control reactions were also performed under identical conditions without hydrogen peroxide and enzyme. The influence of pH on dye degradation was assessed using the optimal biocatalyst concentration and reaction conditions (dye solution concentration and temperature) that had previously yielded the best results

### 2.4 Mathematical Data Treatment

Spectral absorbance data at the maximum absorbance wavelength obtained from UV–vis analysis at pre-established time intervals were used to calculate the dye concentration (represented by *C*_o_ (g L^–1^)) in the liquid phase through interpolation from the analytical curve of Reactive Blue 171.

These data were used in an Excel spreadsheet (Microsoft Windows Seven) to determine the parameters of the dye degradation process (or decolorization of dye solution). Graphic plotting and interpretations were carried out with the Origin 6.1 software.

Activation parameters, including activation energy (*E*_a_), activation enthalpy (Δ*H* ^#^), activation entropy (Δ*S* ^#^), and Gibbs free energy of activation (Δ*G* ^#^), as well as the process rate constants, were determined through eqs 1 to 4 described in the work.

Kinetics showed a first-order behavior, and eq 1 was used to determine the rate constants, in which *C*_0_, initial dye concentration; *C*_*t*_, concentration at time *t*; *k*, rate constant; and *t*, time.^32–34^

$$\ln\;C_{t}=-kt+\ln\;C_{0}$$ 1

The linearized Arrhenius equation was used to calculate the activation energy (eq 2), in which *k*, rate constant; *A*, frequency factor; *E*_a_, activation energy; *R*, gas constant; and *T* (K), absolute temperature

$$\ln\;k=\ln\;A-E_{a}/RT$$ 2

Activation enthalpy and activation entropy were calculated using the Eyring equation (eq 3), where *k*_b_ and *h* are Boltzmann’s and Planck’s constants, respectively.^32–34^

$$\ln(k_{\mathrm{obs}}/T)=\ln(k_{b}/h)+\Delta S^{\#}/R-\Delta H^{\#}/RT$$ 3

The free activation energy (Δ*G* ^#^) was determined using the eq 4, at a *T* equal to 298.15 K

$$\Delta G^{\#}=\Delta H^{\#}-\Delta S^{\#}$$ 4

## 3 Results and Discussion

### 3.1 Initial Evaluation of the Activity of HRP PeO 906/H_2_O_2_ in the Degradation of the Dye Reactive Blue 171

It was observed that the HRP PeO 906 enzyme exhibits maximum activity at approximately 50 °C. At this temperature, the disappearance of the absorption band of the Reactive Blue 171 dye at 625 nm was monitored. To activate the enzyme, a 0.3% hydrogen peroxide solution was used as an oxidant and activating substrate. Figure 2 illustrates the behavior of the absorption band over time. HRP in combination with H_2_O_2_ degrades Reactive Blue 171, resulting in the disappearance of the color of the dye. This is evidenced by the decrease in the absorption band, as shown in Figure 2, during a monitoring period of 120 min.

**Figure 2:**
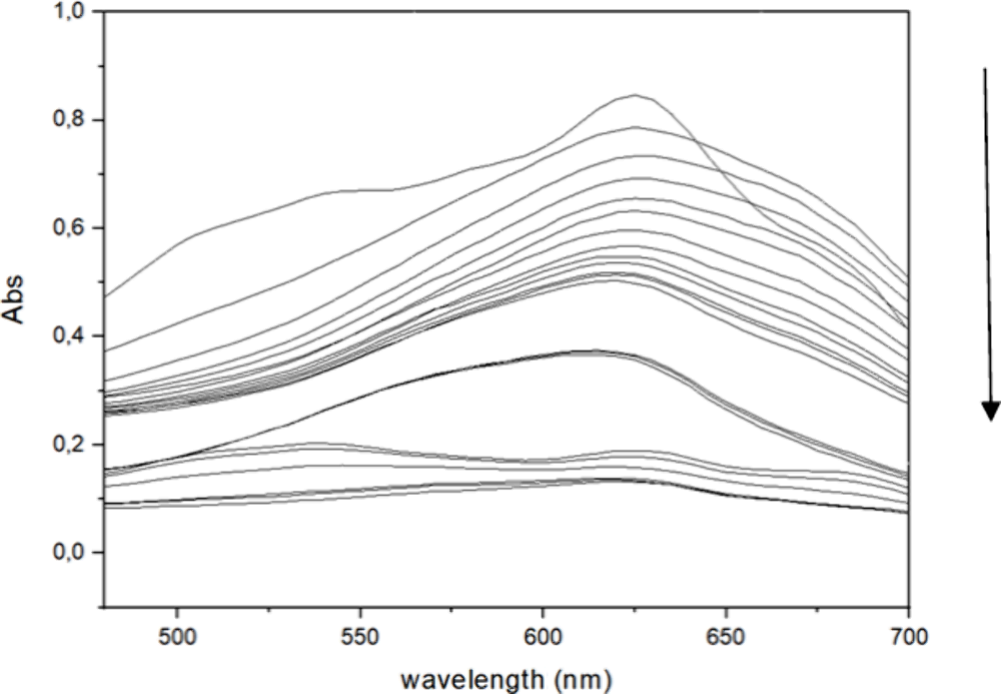
Decrease in the absorption band of Reactive Blue 171 dye catalyzed by HRP PeO 906 peroxidase as a function of time at 50 °C, with [dye] = 0.1 g L^–1^, [H_2_O_2_] = 0.3% (1 mL), and [enzyme] = 3 mg.

Figure 3 shows that HRP PeO 906, in combination with H_2_O_2_, effectively degrades Reactive Blue 171 dye, as indicated by the color loss and an intensity decrease in its absorption band. The influence of the concentrations of hydrogen peroxide and HRP on the degradation of Reactive Blue 171 was examined. The results show that neither the enzyme nor hydrogen peroxide alone induced any decolorization of the dye solution, even with increasing temperature. This confirms that HRP requires the presence of H_2_O_2_ to catalyze dye degradation, with H_2_O_2_ acting as an essential substrate for the reaction.

**Figure 3:**
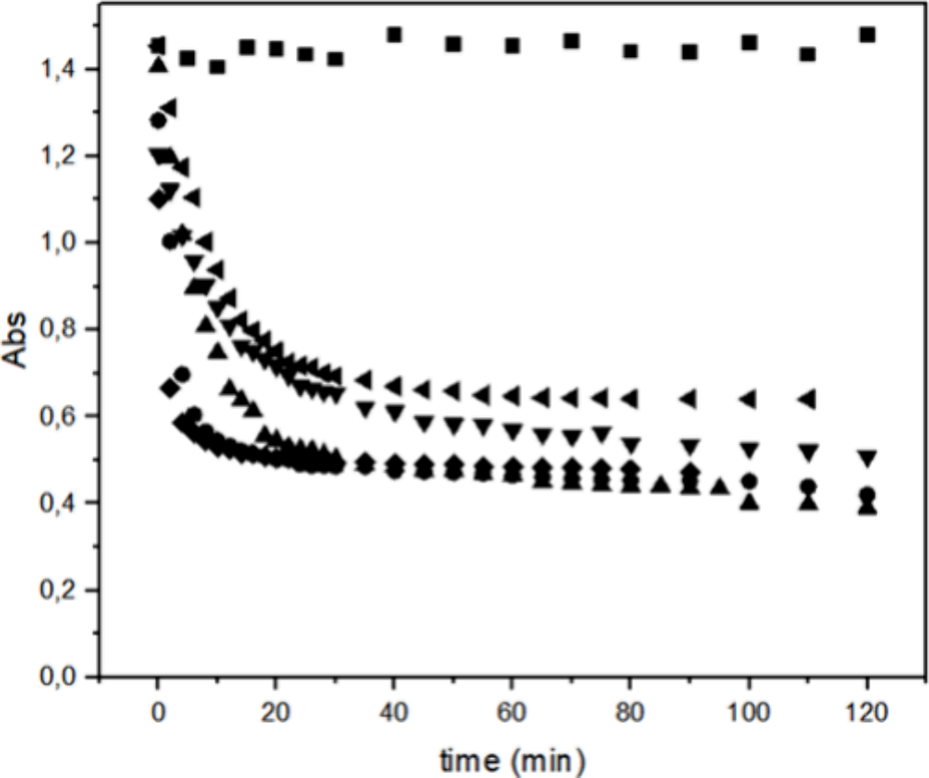
Decolorization of Reactive Blue 171 dye solution with 1 mg of HPR PeO 906 at different temperatures: (■) = 70 °C only in the presence of H_2_O_2_; (●) = 30 °C; (▲) = 40 °C; (▼) = 50 °C; (◆) = 60 °C; (◀) = 70 °C; [H_2_O_2_] = 0.3% and [dye] = 0.1 g L^–1^.

### 3.2 Effect of Enzyme Concentration and Temperature

Kinetic studies were performed by varying the added amount of enzyme from 1 mg to 2 and 3 mg (Figures 4–6, respectively) and assessing the decolorization process at different temperatures (30, 40, 50, 60, and 70 °C), while maintaining a constant hydrogen peroxide concentration (0.3%).

**Figure 4:**
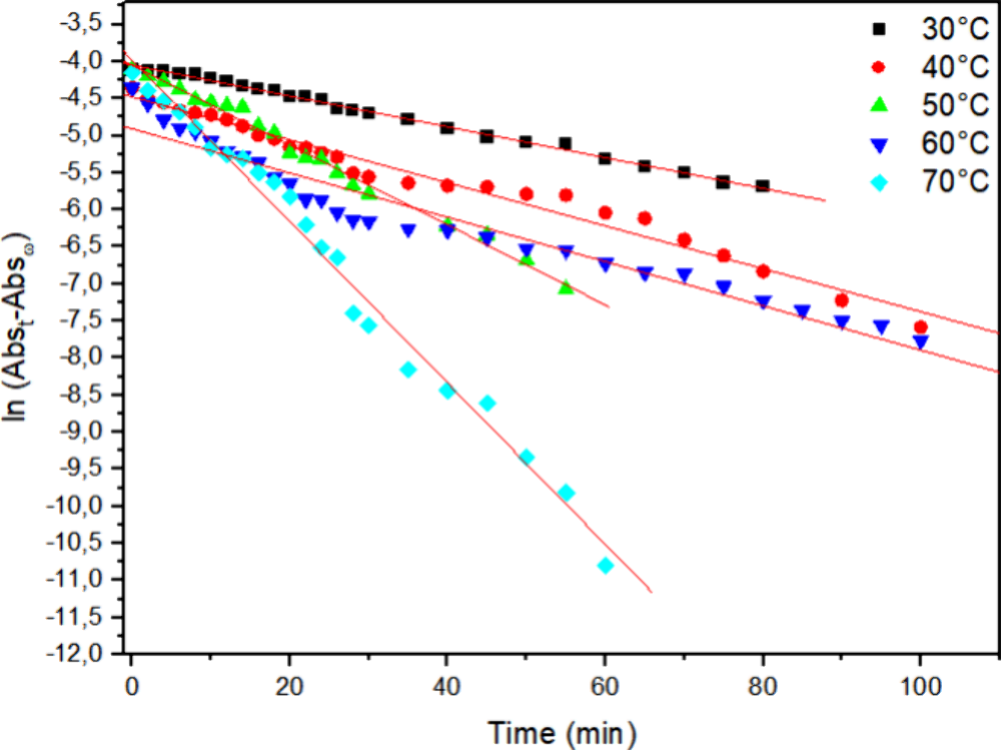
Kinetic curve of decolorization of Reactive Blue 171 solution with 1 mg of HRP PeO 906 at different temperatures: (■) = 30 °C; (●) = 40 °C; (▲) = 50 °C; (▼) = 60 °C; (◀) = 70 °C; [H_2_O_2_] = 0.3% and [dye] = 0.1 g L^–1^.

**5:**
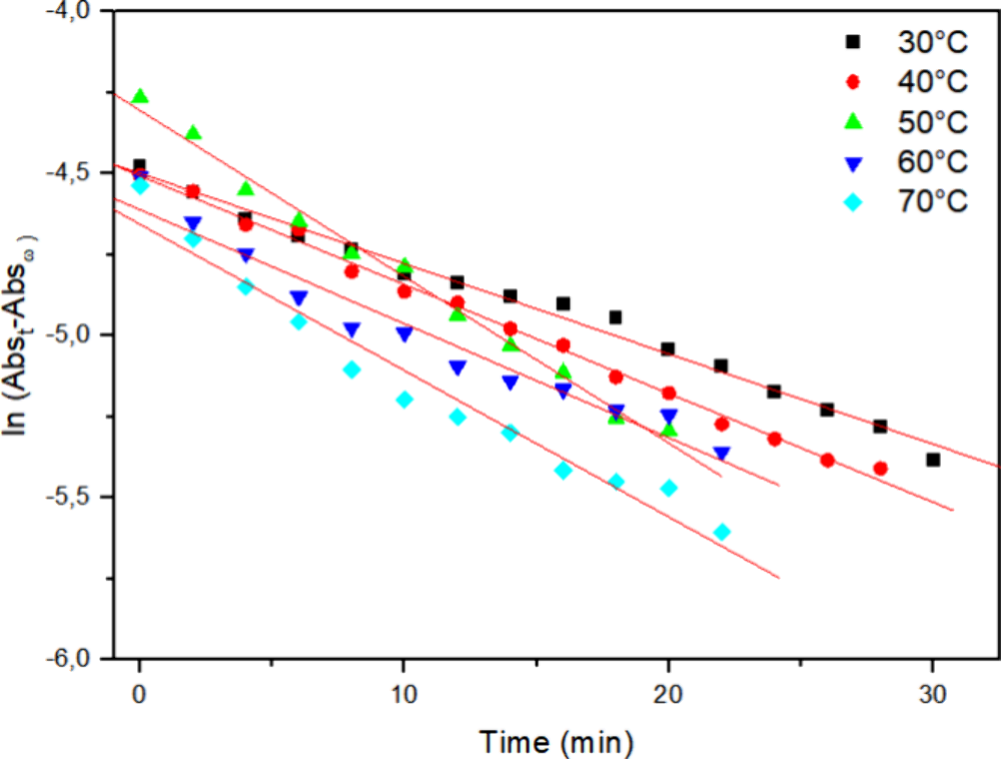
Kinetic curve of Reactive Blue 171 dye decolorization with 2 mg of HRP PeO 906 at different temperatures: (■) = 30 °C; (●) = 40 °C; (▲) = 50 °C; (▼) = 60 °C; (◀) = 70 °C; [H_2_O_2_] = 0.3% and [dye] = 0.1 g L^–1^.

**Figure 6:**
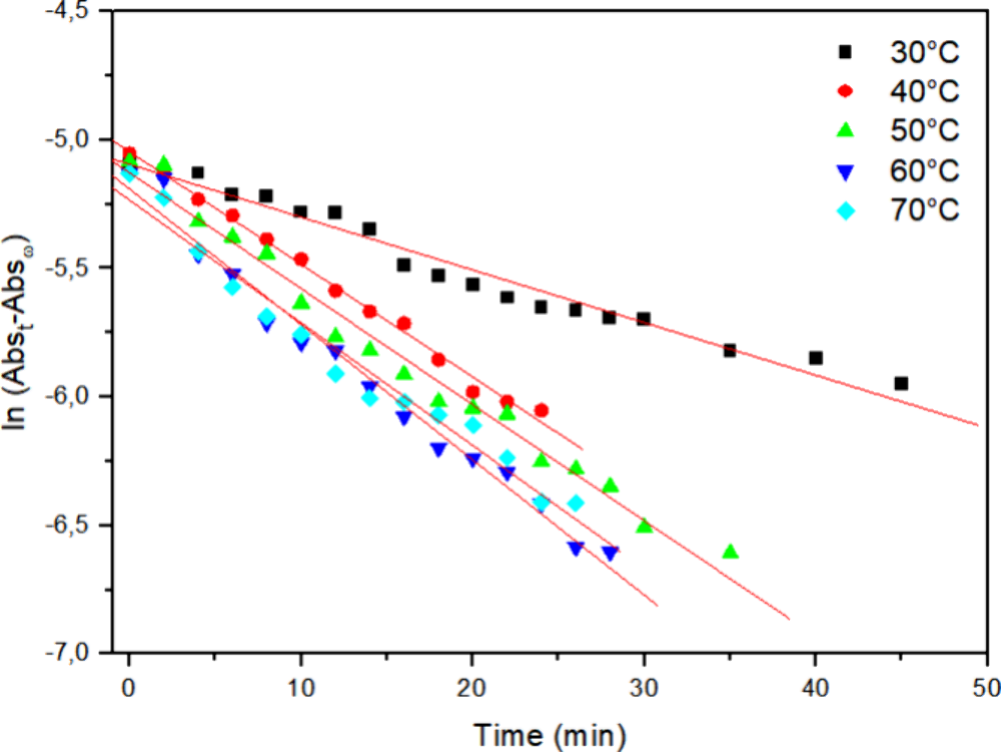
Kinetic curve of Reactive Blue 171 dye decolorization with 3 mg of HRP PeO 906 at different temperatures: (■) = 30 °C; (●) = 40 °C; (▲) = 50 °C; (▼) = 60 °C; (◀) = 70 °C; [H_2_O_2_] = 0.3% and [dye] = 0.1 g L^–1^.

The results indicate that increasing the temperature enhances the dye degradation process. The kinetic analysis of the curves revealed a first-order behavior. Using eq 1, the observed rate constants (*k*_obs_) for degradation (decolorization) at different temperatures were calculated.

In Figures 4–6 ln­[Abs­(*t*) – Abs­(*f*)] was plotted versus time, allowing us to determine the *k*_obs_ from the slope of each graph. The strong linearity of these plots, with correlation coefficients (*r* ^2^) close to 0.99, confirms the reliability of the kinetic model. Table 1 further illustrates a significant increase in *k*_obs_ with increasing temperature, while increasing the added enzyme amount from 1 mg to 2 or even 3 mg did not lead to further decolorization enhancement; instead, even lower *k*_obs_ and higher *t*_1/2_ were observed, which may be eventually attributed to insufficient activation through hydrogen peroxide.

**Table 1:** Effect of Temperature on the Rate Constant for the Decolorization of Reactive Blue 171 Solutions in 120 min

enzyme	1 mg			2 mg			3 mg		
*T*	*t*_1/2_	*k*_obs_	decolorization	*t*_1/2_	*k*_obs_	decolorization	*t*_1/2_	*k*_obs_	decolorization
K	min	min^–1^ 10^–2^	%	min	min^–1^ 10^–2^	%	min	min^–1^ 10^–2^	%
303	21	3.32	67.3	31	2.27	75.2	45	1.5	76.1
313	16	4.33	72.2	27	2.60	74.1	31	2.24	81.4
323	14	4.81	57.7	26	2.66	73.7	27	2.58	76.8
333	12	5.77	66.2	19	3.64	72.3	22	3.19	71.6
343	9	7.51	55.9	17	3.94	72.9	20	3.42	76.5

### 3.3 Activation Parameter Calculations

Using the rate constants obtained at different temperatures and enzyme concentrations, the activation energy (*E*_a_) for the biocatalytic degradation of Reactive Blue 171 was determined by applying the linearized Arrhenius equation (eq 3). A plot of ln *k* versus 1/*T* yielded a straight line with a slope of −*E*_a_/*R*.

Under the same conditions, the variations in activation enthalpy (Δ*H* ^#^), activation entropy (Δ*S* ^#^), and activation free energy (Δ*G* ^#^) were also calculated for the different temperatures used. The values of *E*_a_, Δ*G* ^#^, Δ*H* ^#^, and Δ*S* ^#^ are presented in Table 2.

**Table 2:** Activation Parameters Determined for the Decolorization of Reactive Blue 171

HRP PeO 906 (mg)	*E*_a_ (kJ mol^–1^) (*r* ^2^)	Δ*H* ^#^ (kJ mol^–1^) (*r* ^2^)	Δ*G* ^#^ average (kJ mol^–1^)	Δ*S* ^#^ average (J K^–1^ mol)
1	25.59 (0.9934)	28.56 (0.9950)	16.41	1.89
2	25.31 (0.9413)	27.98 (0.9373)	16.10	0.992
3	13.24 (0.9919)	10.55 (0.9717)	14.09	0.109

The positive values of Δ*H* ^#^, Δ*G* ^#^, and Δ*S* ^#^ confirm that the oxidative degradation of Reactive Blue 171 is an endothermic process. The *E*_a_ and Δ*H* ^#^ values indicate that the reaction occurs with a low heat content, while the low Δ*S* ^#^ values suggest an organized reaction mechanism.

### 3.4 Influence of pH on the Biodegradation of Reactive Blue 171 Dye

To evaluate the influence of pH, McIlvaine buffer solutions were prepared at pH values of 4, 5, 6, 7, and 8. The experiments were conducted under the following conditions: 0.03% hydrogen peroxide, 0.1 g L^–1^ dye concentration, and a temperature of 40 °C, while varying the enzyme concentration 1, 2, and 3 mg (Figures 7–9, respectively). These figures illustrate the decrease in absorbance over time for the different pH conditions.

**Figure 7:**
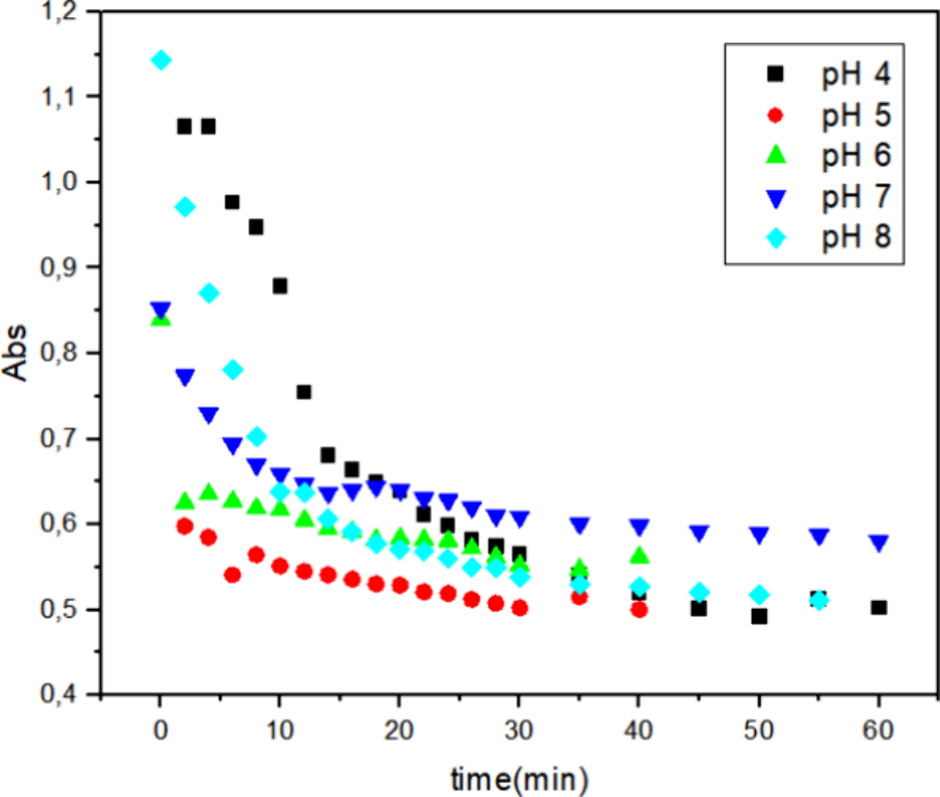
Decolorization of 0.1 g L^–1^ Reactive Blue 171 solution with 1 mg of HRP PeO 906 and 0.03% H_2_O_2_ at 40 °C under different pH values: (■) = pH 4; (●) = pH 5; (▲) = pH 6; (▼) = pH 7; (◆) = pH 8; [H_2_O_2_] = 0.03% and [dye] = 0.1 g L^–1^.

With 1 mg of HRP PeO 906 (Figure 7), at pH 5, the highest degradation efficiency was observed, with a rapid decrease in absorbance, indicating effective dye degradation. At pH 4, the degradation efficiency is slightly lower than that at pH 5, but still relatively high. At pH 6, there is a noticeable decrease in the degradation efficiency. At pH 7, there was a further decrease in the degradation efficiency, indicating reduced enzyme activity under neutral conditions. At pH 8, there was a slight improvement in the degradation efficiency compared to pH 7, but it was still lower than that at pH 5.

With 2 mg of HRP PeO 906 (Figure 8), at pH 5, the highest degradation efficiency, similar to that of the 1 mg enzyme concentration but with a more pronounced decrease in absorbance, was obtained. At pH 4, the degradation efficiency was high but slightly lower than that at pH 5. At pH 6, an improved degradation efficiency compared to the 1 mg enzyme concentration was obtained. At pH 7, better degradation efficiency than that with 1 mg enzyme was obtained, but it was still lower than that in acidic conditions. At pH 8, the degradation efficiency improved compared to that of 1 mg enzyme, but it was lower than that at pH 5.

**Figure 8:**
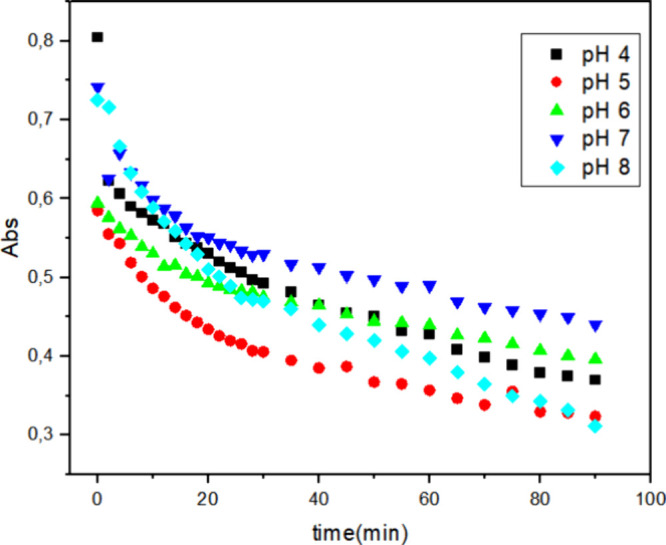
Decolorization of 0.1 g L^–1^ Reactive Blue 171 solution with 2 mg of HRP PeO 906 and 0.03% H_2_O_2_ at 40 °C at different pH values: (■) = pH 4; (●) = pH 5; (▲) = pH 6; (▼) = pH 7; (◆) = pH 8; [H_2_O_2_] = 0.03% and [dye] = 0.1 g L^–1^.

With 3 mg of HRP PeO 906 (Figure 9), at pH 5, the highest degradation efficiency was observed, with the most rapid decrease in absorbance. At pH 4, a high decolorization efficiency, but slightly lower than that at pH 5, was observed. At pH 6, the degradation efficiency significantly improved compared to that at lower enzyme concentrations. At pH 7, better degradation efficiency than those with 1 and 2 mg enzyme was observed, but it was still lower than that in acidic conditions. At pH 8, the degradation efficiency improved compared to that at lower enzyme concentrations, but it was lower than that at pH 5. The decolorization efficiency was further enhanced across all pH levels.

**Figure 9:**
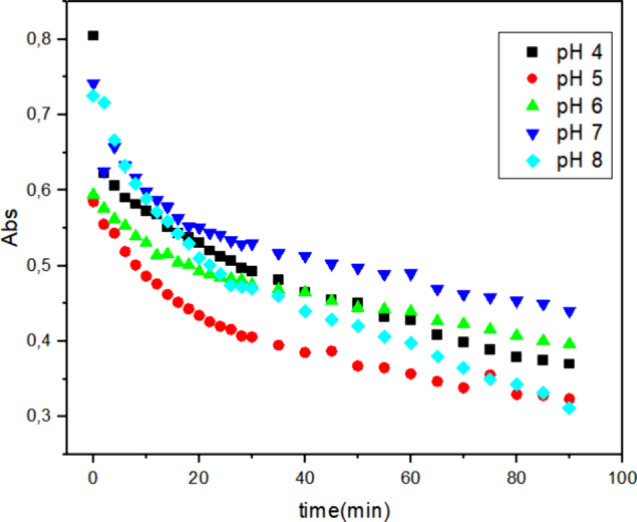
Decolorization of 0.1 g L^–1^ Reactive Blue 171 solution with 3 mg of HRP PeO 906 and 0.03% H_2_O_2_ at 40 °C at different pH values: (■) = pH 4; (●) = pH 5; (▲) = pH 6; (▼) = pH 7; (◆) = pH 8; [H_2_O_2_] = 0.03% and [dye] = 0.1 g L^–1^.

The observed rate constants (*k*_obs_) obtained from these experiments are summarized in Table 3. It showed that both pH and enzyme concentration significantly influence the degradation efficiency of the Reactive Blue 171 dye. The enzyme exhibits optimal catalytic activity under acidic conditions, particularly at pH 4. An increase in the enzyme concentration enhances the degradation efficiency across all pH levels, with the highest efficiency observed at 3 mg of enzyme. These findings highlight the importance of optimizing both pH and enzyme concentration to achieve the maximum degradation efficiency for the biocatalytic treatment of dye-contaminated wastewater.

**Table 3:** Kinetic Parameters for the Decolorization of Buffered Reactive Blue 171 Solutions over 120 min

1 mg de enzyme				2 mg de enzyme			3 mg de enzyme		
	*t*_1/2_	*k*_obs_	decolorization	*t*_1/2_	*k*_obs_	decolorization	*t*_1/2_	*k*_obs_	decolorization
pH	min	min^–1^ 10^–2^	%	min	min^–1^ 10^–2^	%	min	min^–1^ 10^–2^	%
4	4	16.64	66.6	7	9.24	75.1	3	2.274	74.1
5	5	14.71	65.8	8	8.84	73.2	5	2.875	78.8
6	7	10.04	62.0	9	7.37	66.8	6	1.713	73.5
7	15	4.57	60.9	10	7.27	62.3	11	1.429	78.2
8	16	4.23	66.3	17	4.11	62	12	5.870	70.1

### 3.5 Comparison with Other Studies

Table 4 summarizes previous studies on the degradation of the Reactive Blue 171 dye.

**Table 4:** Degradation of the Reactive Blue 171 Dye as Reported in Previous Studies

reference	method	degradation
Patil and Shukla^35^	UV/H_2_O_2_	2%
Patil and Shukla^35^	ozonation	33%
Patil and Shukla^36^	Oxidation using ceric ammonium nitrate.	93.1%
Mahroudi et al.^37^	peroxi-electrocoagulation process, pH 3, 9.2 mA cm^–2^ current density, 2.5 cm distance between the electrodes, 4 mM H_2_O_2_, and 7.7 min reaction time.	99.4%
Bakar et al.^38^	laccase from *Trametes trogii* catalyzed by Zr-based metal–organic framework (MOF) at pH 4.5 and 60 °C.	56.7%
Vantamuri and Kaliwal^39^	White rot fungus *Marasmius sp*. BBKAV79, 50 mg L^–l^, within 24 h under shaking condition.	100% using urea and NH_4_Cl as the nitrogen source
Suliman et al.^40^	Sunlight irradiation catalyzed by TiO_2_ doped with Fe_2_O_3_, concentration from 1 to 3 mg L^–1^.	100% in 2 h
Boran^41^	Yeast immobilized cotton-based structures were used for color removal of Reactive Blue 171.	55–57% in 24 h
This study	HRP-catalyzed degradation of 0.1 g L^–1^ dye at 40 °C, using 2 mg of HRP and 0.3% H_2_O_2_.	81.4%

Patil and Shukla^35^ were unable to degrade the dye using only UV and H_2_O_2_, while ozonation resulted in a low degradation efficiency. Dye degradation using ceric ammonium nitrate was effective but costly and generated cerium waste.^36^ The peroxi-electrocoagulation process achieved high degradation levels; however, it was expensive due to high electricity consumption.^37^

The method described by Bakar et al.^38^ using laccase in MOF achieved only 56.7% decolorization, while the approach by Vantamuri and Kaliwal^39^ required 24 h and the addition of a nitrogen source, leading to increased waste. Suliman et al.^40^ reported high degradation using only sunlight, but their study used dye concentrations 100 times lower than those used in this research. Similarly, the study by Boran^41^ resulted in lower degradation, even with an extended treatment time of 24 h.

In contrast, this study achieved 59.8% dye degradation at ambient temperature for a relatively high dye concentration (100 mg L^–1^) and by using only 1 mg of HRP in just 2 h, demonstrating a more efficient and environmentally friendly approach.

## 4 Conclusions

This study demonstrated that HRP PeO 906, in combination with H_2_O_2_, effectively catalyzes the degradation of the Reactive Blue 171 dye. The degradation process followed first-order kinetics, with the rate constant increasing with temperature. The calculated activation parameters, including activation energy, enthalpy, entropy, and Gibbs free energy, indicated that the degradation required low energy input and involved minimal structural changes in the transition state.

The optimal conditions for dye degradation were found to be with a dye concentration of 0.1 g L^–1^ at 40 °C, using 3 mg of enzyme together with 0.3% H_2_O_2_, achieving 81.4% decolorization within 120 min. pH studies revealed that the highest dye degradation occurred at pH 4, with significant degradation also observed at pH 8.

In comparison to existing and previously reported wastewater decolorization methods, the HRP PeO 906/H_2_O_2_ system offers a cost-effective, efficient, environmentally friendly, and green approach for treating dye-contaminated wastewater. This enzymatic degradation process minimizes chemical waste and operates under mild conditions, making it a promising alternative to traditional physicochemical treatments. Future studies should focus on improving the enzyme stability, optimizing reaction conditions for large-scale applications, and exploring the degradation of structurally diverse dyes using similar biocatalytic systems.
